# Cigarette smoke-induced circFOXO3 upregulation enhances autophagy-regulated senescence of type II alveolar cells through interacting with E2F1

**DOI:** 10.18332/tid/215393

**Published:** 2026-01-24

**Authors:** Xia Zhou, Yun Wang, Jinchang Lu, Bing Wang, Feng Zhou, Jing Pan, Lei Zhou, Chunling Du

**Affiliations:** 1 Department of Pulmonary and Critical Care Medicine, QingPu Branch of Zhongshan Hospital Affiliated to Fudan University, Shanghai, China; 2 Shanghai Key Laboratory of Medical Internet of Things, Shanghai, China

**Keywords:** chronic obstructive pulmonary disease, circular RNA FOXO3, E2F1, senescence, autophagy

## Abstract

**INTRODUCTION:**

Senescence of type II alveolar (AT-II) cells is involved in the pathogenesis of chronic obstructive pulmonary disease (COPD). We have reported that circRNA FOXO3 (circFOXO3) is upregulated after exposure to cigarette smoke (CS) and that circFOXO3 knockdown has protective effects on CS-induced inflammation. Here, we investigate whether circFOXO3 upregulation is involved in CS-induced AT-II cell senescence.

**METHODS:**

Within this experimental cell-based and animal study, the effects of circFOXO3 on CSE-induced senescence in AT-II cell line MLE12 were determined by senescence-associated β-galactosidase staining and western blotting analyses of p16 and p21 expression. Immunofluorescence staining was used to determine the expression of γ-H2AX to analyze DNA damage. Then the autophagy level of CSE-treated MLE12 cells was evaluated by western blotting analyses of LC3B and Beclin-1 expression. Furthermore, we analyzed interactions between circFOXO3 and E2F transcription factor 1 (E2F1) in RNA binding protein immunoprecipitation studies.

**RESULTS:**

Our results show that circFOXO3 knockdown suppressed CS extract (CSE)-induced senescence in the AT-II cell line MLE-12. Additionally, CSE-induced autophagy impairment was reduced by circFOXO3 knockdown, and the autophagy inhibitor 3-methyladenine abrogated the effects induced by circFOXO3 knockdown on cell senescence. Mechanistic investigations revealed that circFOXO3 interacts with E2F1 and suppresses its nuclear translocation. E2F1 knockdown reduced the positive regulation of circFOXO3 knockdown on autophagy and prevented the suppressive effects of circFOXO3 knockdown on cell senescence. Consistent with this, circFOXO3 knockdown mitigated CS-induced senescence in AT-II cells *in vivo*.

**CONCLUSIONS:**

Overall, these findings suggest that CS-induced circFOXO3 upregulation promotes autophagy-dependent senescence of AT-II cells, leading to enhanced lung injury.

## INTRODUCTION

Chronic obstructive pulmonary disease (COPD) is a serious public health issue and is characterized by destruction of the alveolar wall, inflammation, premature lung aging, and cellular senescence^1,2^. It is well established that cigarette smoke (CS) is a major risk factor that causes and accelerates COPD. Recent evidence has also highlighted the critical role of cellular senescence, particularly of type II alveolar (AT-II) cells, in the progression of COPD^3,4^. AT-II cells secrete many types of pulmonary surfactant proteins, which promote lung repair, while AT-II cell senescence initiates communication with other cells and eventually causes lung injury^5,6^. Thus, inhibition of AT-II cell senescence may be an effective prevention and treatment strategy for COPD.

Circular RNAs (circRNAs) are a class of non-coding RNA with a special closed-loop structure^7,8^. CircRNAs are widely expressed in eukaryotes and regulate genes by interacting with RNA and/or proteins^9^. Recent evidence has shown that abnormal circRNA expression is involved in various human diseases^10^. A number of recent studies have indicated that circRNAs such as circ0061052, circANKRD11, circRNA_0026344, and circ-HACE1 may also exert crucial functions in CS-induced lung injury^11-14^. CircRNA FOXO3 (circFOXO3) is a conventional exonic circRNA involved in the regulation of several disorders, including cardiac senescence, blood-brain barrier damage, and cancers^15-17^. We previously demonstrated that circFOXO3 is highly expressed after CS treatment, and its knockdown has a protective effect against lung inflammation^18^. However, how circFOXO3 is involved in CS-induced AT-II cell senescence remains largely unknown.

Autophagy is a universal, dynamic process whereby changes in the cellular environment result in cytoplasmic materials, including soluble macromolecules and organelles, being transported to lysosomes for degradation. Therefore, autophagy can mediate cell damage and is associated with many human diseases^19^. Recent evidence suggests that autophagy plays an indispensable role in lung epithelial injury caused by CS^19,20^. Abnormal autophagy in the lungs may lead to CS-induced cell senescence and lung aging^21^. Restoration of autophagy in AT-II cells inhibits CS-related cell senescence and pulmonary fibrosis^6^.

This study investigated the potential function of circFOXO3 in CS-induced senescence of AT-II cells. We propose the hypothesis that circFOXO3 knockdown may suppress CSE-induced senescence in MLE-12 cells by activating autophagy.

## METHODS

This is an experimental cell-based and animal study aimed at exploring the functional mechanisms of circFOXO3 in CS-induced senescence of AT-II cells.

### Cell-based study

*Cell culture*

MLE-12 cells (a mouse AT-II cell line) were grown in DMEM + 10% FBS (Gibco, Grand Island, NY, USA) at 37°C in a 5% CO_2_ atmosphere. For lentiviral transduction, MLE-12 cells were infected with circFOXO3 knockdown or overexpression lentivirus in the presence of 8 mg/mL polybrene. For CSE treatment, infected cells were treated with 2.5% CSE, and the cells were harvested after 48 h for analysis.

*CSE preparation*

CSE was prepared as previously described^18^. Five cigarettes were obtained from Shanghai Double Happiness (Shanghai, China) and their smoke was bubbled through DMEM medium (40 mL). The solution was then passed through a 0.22 μm filter and defined as a 100% concentration of CSE. In subsequent assays, this solution was diluted with fresh DMEM to a final concentration of 2.5% before each experiment.

*Quantitative real-time PCR (qPCR)*

RNA was extracted by use of TRIzol^®^ Reagent (Sigma-Aldrich, St. Louis, MO, USA), and 1 mg of total RNA was used for reverse transcription using oligo-dT primers. Real-time PCR was then performed using SYBR™ Green PCR Master Mix (Applied Biosystems, Foster City, CA, USA). The relative expression of circFOXO3 was normalized to that of *GAPDH* and quantitated using the 2^-ΔΔCT^ method.

*Western blotting*

Cells were subjected to protein extraction using RIPA lysis buffer (Beyotime Biotechnology, Shanghai, China), and the protein extracts were subjected to western blotting as previously described^18^. The primary antibodies used in this study were anti-p16, anti-p21, anti-LC3B, anti-E2F1 (Abcam, Cambridge, UK), anti-Beclin 1, anti-PCNA, anti-SFTPC, and anti-GAPDH (Proteintech, Wuhan, China).

*Cell senescence-associated β-galactosidase staining (SA-β-gal)*

This assay was performed according to the instructions of the β-gal staining kit (Beyotime Biotechnology). Cells in 12-well plates were washed twice with PBS, fixed, and then incubated in the staining solution overnight at 37°C. The ratio of positive cells was analyzed.

*Immunofluorescence staining and mitochondrial superoxide detection*

Immunofluorescence staining was performed as previously described^22^. Lung sections were deparaffinized, followed by antigen retrieval through heat treatment in citrate buffer. Cell climbing slices were prepared for standard immunofluorescence. The samples or cell climbing slices were blocked with 5% goat serum and then incubated with primary antibodies against p16, p21 (Abcam), SFTPC and γH2AX (Proteintech) overnight at 4°C, followed by incubation with secondary antibodies and staining with DAPI. Finally, the stained cells were imaged with a fluorescence microscope (Nikon, Japan). For mitochondrial superoxide detection, cells were incubated with mitoSOX Red (2 μM) for 10 min at 37°C in the dark. Following washing with warm HBSS/Ca/Mg, cells were captured by a fluorescence microscope.

*RNA binding protein immunoprecipitation (RIP)*

A Magna RIP^TM^ RNA-binding Protein Immunoprecipitation Kit (Millipore, MA, USA) was used to perform the RIP assay^23^. Briefly, 5 μg of E2F1 antibody (Abcam) or normal IgG (Millipore) in 500 μL of lysis buffer containing protease inhibitor cocktail was immobilized on magnetic beads by incubation for 60 min at 37°C. Cell lysates were then prepared in complete lysis buffer and added to tubes containing the antibody-coated beads and incubated overnight at 4°C. RNA-protein complexes were used to purify RNA, followed by qPCR analysis to detect the enrichment of circFOXO3.

*circRNA pull-down assays*

This pull-down assay was performed as previously described^15^. Cells were lysed in complete lysis buffer, followed by incubation overnight at 37°C with biotin-labeled probes. Streptavidin magnetic beads were then added to the cell lysates and incubated for 60 min at 37°C. The beads were magnetically separated and washed six times. RNA-protein complexes were used to extract proteins, which were then subjected to western blot analysis.

### Animal studies

C57BL/6 mice (male, 6 weeks old) were obtained from SLAC (Shanghai, China). The mice in the CS model group were subjected to full-body CS exposure, according to previously described methods^12,13^. These mice were exposed to CS from five cigarettes twice a day, five days a week, for 12 weeks. The mice in the control group were kept in ambient air. For lentivirus treatment, circFOXO3 knockdown lentiviruses were injected into the tail vein of mice once every two weeks after the first CS exposure. Each group included six mice. All *in vivo* manipulations were approved by the Animal Care and Use Committee of Shanghai Chengxi Biotechnology Co., Ltd, and proceeded in accordance with the ARRIVE Guidelines for the Care and Use of Laboratory Animals.

### Statistical analysis

The statistical analyses were performed using GraphPad Prism version 5 software. Data are presented as mean ± standard deviation of at least 3 independent experiments. The significance was determined using Student’s t-test when comparing only two groups or assessed by one-way analysis of variance when more than two groups were compared. Statistical significance was set at p<0.05.

## RESULTS

### Downregulation of circFOXO3 suppressed CSE-induced senescence in AT-II cells

**Figure 1:**
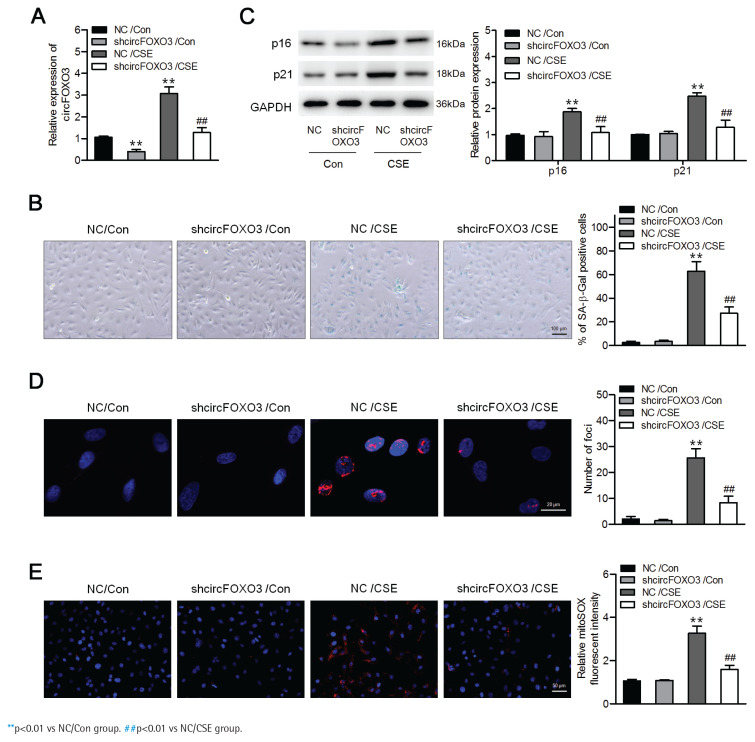
Downregulation of circFOXO3 suppressed CSE-induced senescence in MLE12 cells: A) MLE12 cells were infected with lentivirus carrying circFOXO3 shRNA (shcircFOXO3) or NC, and qPCR was used to determine circFOXO3 levels in these cells treated with or without 2.5% CSE for 24 h (n=3); B) SA-β-gal staining of the indicated cells (n=3); C) Western blotting was used to determine the expression levels of p16 and p21 in cells treated with or without 2.5% CSE (n=3); D) Immunofluorescence microscopy of γH2AX in the indicated cells (n=3); E) MitoSOX™ Red staining (n=3)

To determine the effects of circFOXO3 on CSE-induced senescence in AT-II cells, we first infected the mouse AT-II cell line MLE12 with a lentivirus carrying circFOXO3 shRNA, and a significant decrease in circFOXO3 expression was verified by qPCR (Figure 1A). In addition, Figure 1B shows that CSE treatment significantly increased the number of senescent cells, based on the increased proportion of SA-β-gal-positive cells, whereas circFOXO3 knockdown reversed this increase. Accordingly, circFOXO3 knockdown markedly reduced the CSE-induced expression of p16 and p21 (Figure 1C). Next, we used immunofluorescence staining to determine the expression of γ-H2AX to analyze DNA damage, and the results showed that circFOXO3 knockdown attenuated the increase in γH2AX foci formation induced by CSE (Figure 1D). We also showed that mitochondria-derived ROS (mitoROS) levels were elevated by CSE, whereas circFOXO3 knockdown decreased mitoROS levels (Figure 1E). These results demonstrate that circFOXO3 acts as a promoter for AT-II cell senescence, and downregulation of circFOXO3 reduced CSE-induced cell senescence.

### Downregulation of circFOXO3 enhanced autophagy and reduced CSE-related senescence of AT-II cells

**Figure 2:**
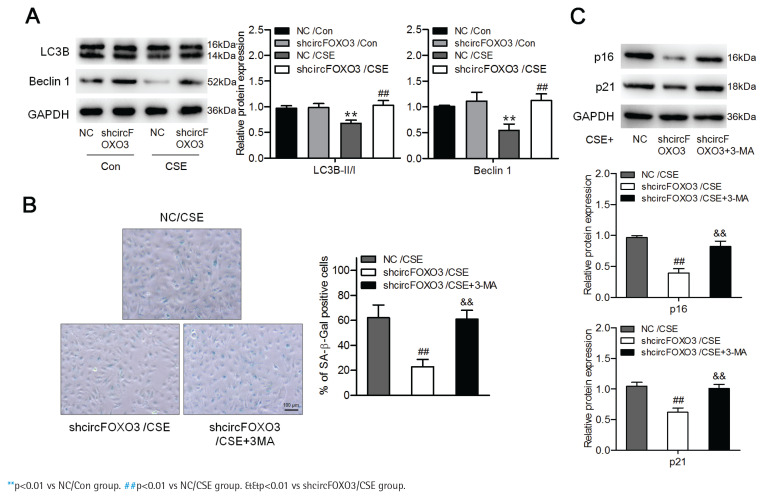
Downregulation of circFOXO3 enhanced autophagy and mediated CSE-related senescence of AT-II cells: A) Western blot analysis of LC3B and Beclin-1 in circFOXO3-knockdown MLE12 cells treated with or without 2.5% CSE for 24 h (n=3); B) SA-β-gal staining of cells treated with 2.5% CSE and/or 3-MA (n=3); C) Western blot analysis of LC3B, p16, and p21 in cells treated with 2.5% CSE and/or 3-MA (n=3)

We further investigated whether circFOXO3 regulates AT-II cell autophagy. To answer this question, we detected the levels of the autophagy-related proteins LC3B and Beclin-1 by Western blotting of lysates of MLE12 cells with knockdown of circFOXO3 and treated with 2.5% CSE. We found that the LC3B-II/I ratio and the Beclin-1 level were reduced by CSE, whereas circFOXO3 knockdown markedly decreased this reduction (Figure 2A). Furthermore, we found that inhibiting autophagy by 3-MA treatment prevented the suppressive effects of circFOXO3 knockdown on cell senescence in the presence of CSE (Figures 2B and 2C). These observations demonstrate that circFOXO3 reduces autophagy and mediates CSE-related cell senescence.

### circFOXO3 interacted with E2F1 and suppressed its nuclear translocation

Next, we tested the potential interactions between circFOXO3 and the senescence-associated protein E2F1. Our results showed that circFOXO3 was pulled down by an antibody against E2F1 in lysates of MLE12 cells treated with 2.5% CSE (Supplementary file Figure S1A). Similar results were obtained with lysates of vector- and circ-FOXO3-transfected cells (Supplementary file Figure S1B). Accordingly, silencing endogenous circFOXO3 significantly decreased the levels of circFOXO3 pulled down by E2F1 (Supplementary file Figure S1C). Furthermore, we performed a pull-down assay using a biotinylated probe, and the results demonstrated that increased amounts of E2F1 were pulled down by the probe compared to the control (Supplementary file Figure S1D). We then examined the effect of circFOXO3 on the expression of E2F1. Supplementary file Figure S1E shows that overexpression or inhibition of circFOXO3 did not affect E2F1 expression; however, the level of E2F1 decreased greatly in the cells overexpressing circFOXO3 overexpression compared with the control cells, while knockdown of endogenous circFOXO3 resulted in nuclear accumulation of E2F1. We also observed enhanced E2F1 expression in the nucleus of the circFOXO3-knockdown cells after CSE treatment (Supplementary file Figure S1F). To further confirm that circFOXO3 knockdown suppressed CSE-induced cell senescence by regulating E2F1 nuclear translocation, we knocked down E2F1 by siRNA in MLE12 cells, and we found that knockdown of E2F1 reduced the effects of circFOXO3 knockdown on autophagy and senescence of AT-II cells (Supplementary file Figures S1G–S1I).

### Downregulation of circFOXO3 attenuated CS-induced AT-II cell senescence in vivo

To further determine the effects of circFOXO3 knockdown on AT-II cell senescence, CS-exposed mice were treated with circFOXO3-knockdown lentivirus. As shown in Supplementary file Figure S2, CS-exposed lungs exhibited an elevated number of β-gal-positive cells compared with the air group. Consistent with this, the numbers of p16- and p21-positive AT-II cells in CS lungs declined, as indicated by immunofluorescence staining (Supplementary file Figures S3 and S4); however, knockdown of circFOXO3 reduced CS-induced AT-II cell senescence. Furthermore, LC3 expression was restored by circFOXO3 knockdown in ATII cells (Supplementary file Figure S5).

## DISCUSSION

There is increasing evidence that certain circRNAs are important regulators of a variety of lung diseases^24-26^. However, much is still unknown regarding their functions in COPD and their effects in regulating the senescence of AT-II cells. In this study, we found that circFOXO3 knockdown suppressed CSE-induced senescence in MLE-12 cells by activating autophagy. In addition, we demonstrate that circFOXO3 interacts with E2F1 and suppresses its nuclear translocation. Finally, we confirmed that circFOXO3 knockdown can mitigate CS-induced autophagy impairment and senescence *in vivo*.

CircFOXO3 is an exonic circRNA that is highly expressed in numerous tissues^15^. Overexpression of circFOXO3 promotes cellular senescence and exacerbates doxorubicin-induced cardiomyopathy^15^. Additionally, circFOXO3 attenuates blood-brain barrier damage during ischemia/reperfusion^16^. Moreover, circFOXO3 relieves myocardial ischemia/ reperfusion injury in myocardial infarction^27^. Our group previously showed that circFOXO3 knockdown ameliorates CS-induced lung injury in mice^18^. As an extension of this study, we here demonstrate that knockdown of circFOXO3 suppressed CSE-induced senescence in MLE-12 cells. Additionally, CSE-induced autophagy impairment was reduced by circFOXO3 knockdown, and 3-MA treatment abrogated the effects induced by circFOXO3 knockdown on cell senescence, suggesting that downregulation of circFOXO3 attenuated CS-related AT-II cells senescence by activating autophagy.

Recent evidence suggests that autophagy plays a pivotal albeit controversial role in lung injury following CS exposure. Impairment of autophagy has been reported to cause cell senescence and pulmonary fibrosis^6,28^. However, other reports have shown that CSE induces autophagy in airway epithelial cells and that autophagy accelerates bronchitis-like airway inflammation^19,20^. Our results are consistent with the former, as we found that CS decreased autophagy to induce AT-II cell senescence. Furthermore, circFOXO3 knockdown reduced autophagy impairment, thus decreasing the CSE-mediated reduction in cell senescence.

E2F1 is a transcription factor that plays central roles in cell proliferation, development, apoptosis, and senescence^29,30^. Disruption of E2F1 function may lead to age-dependent behavioral deficits and synaptic perturbations^29^. Furthermore, E2F1 mediates downregulation of POLD1 during replicative senescence^30^. In this study, we demonstrated that circFOXO3 interacts with the transcription factor E2F1 and suppresses its nuclear translocation. E2F1 knockdown reduced the effects of circFOXO3 knockdown on AT-II cell autophagy and senescence.

Thus, we believe that the function of E2F1 is inhibited in the presence of circFOXO3, thereby promoting cellular senescence.

### Limitations

The present study acknowledges several limitations. Firstly, the primary experiments were conducted using MLE-12 cells, which may not fully capture the complexity of lung tissue *in vivo*. Secondly, the *in vivo* results were derived from mouse models that do not entirely replicate human lung physiology and disease progression. Finally, while this study elucidates the roles of circFOXO3 and E2F1, it is important to note that other pathways involved in COPD and cellular senescence may also play significant roles. Additional factors influencing autophagy and senescence – such as various RNAs, proteins, or environmental variables – require further investigation.

## CONCLUSIONS

Our data demonstrate that CS-induced circFOXO3 upregulation could directly interact with E2F1 and enhance AT-II cell senescence by inhibiting autophagy, providing a novel understanding in the pathogenesis of COPD.

## Supplementary Material



## Data Availability

The data supporting this research are available from the authors on reasonable request.
