# Supplementary material for: Cigarette smoke-induced circFOXO3 upregulation enhances autophagy-regulated senescence of type II alveolar cells through interacting with E2F1
Source: Tob Induc Dis. 2026 Jan 24;24:10.18332/tid/215393. doi: 10.18332/tid/215393 (PMC12831474; doi:10.18332/tid/215393)
Supplement: Supplementary file 1 [file TID-24-09-s1.pdf]

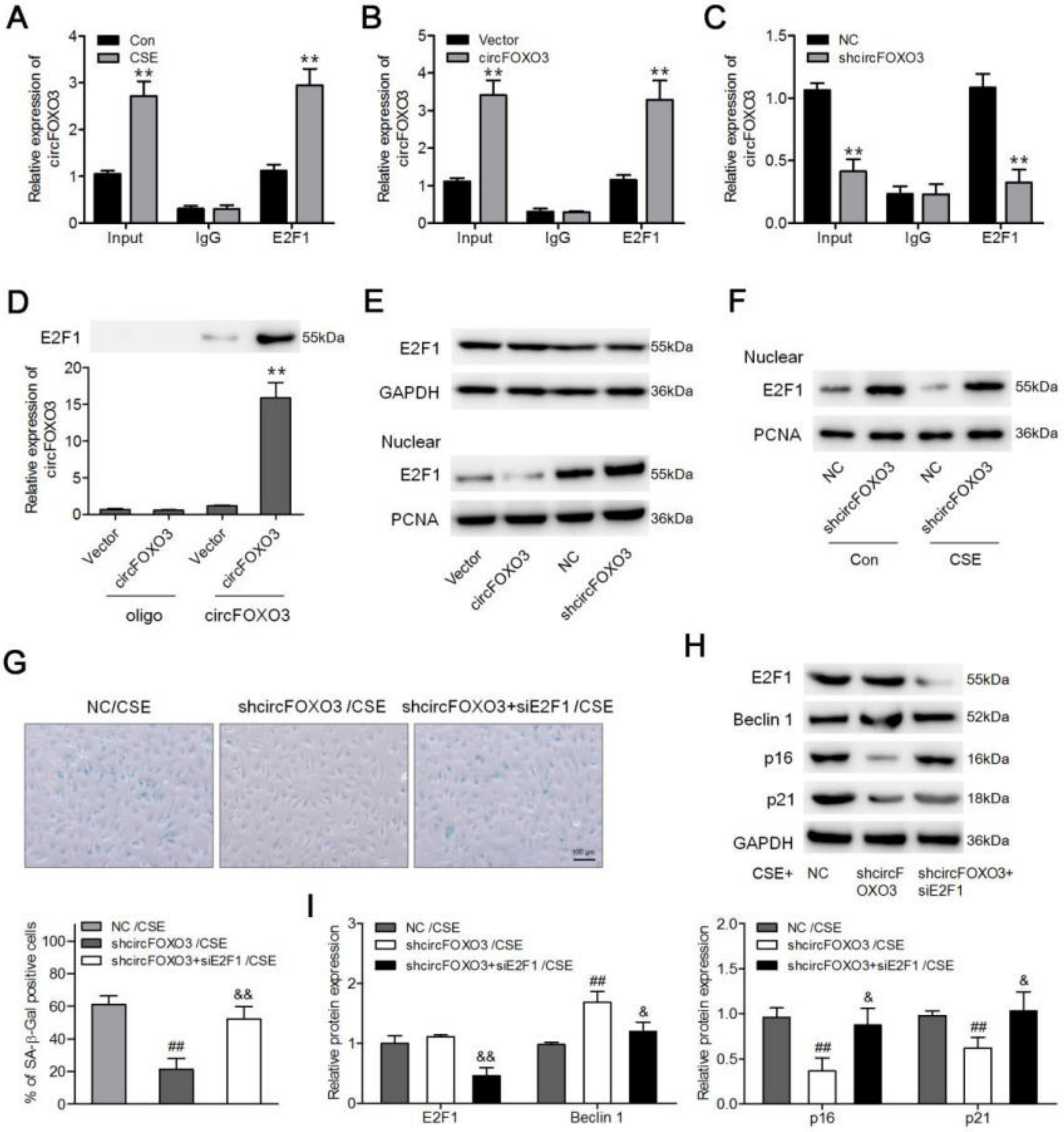

Supplementary Figure S1. CircFOXO3 interacted with E2F1 and suppressed its nuclear translocation. (A) Cell lysates prepared from Con or 2.5% CSE-treated MLE12 cells were subjected to immunoprecipitation with antibodies against mouse IgG or E2F1, followed by qPCR (n = 3). (B) Cell lysates prepared from circFOXO3-overexpressing MLE12 cells were subjected to immunoprecipitation followed by qPCR (n = 3). (C) Cell lysates prepared from circFOXO3-knockdown MLE12 cells were subjected to immunoprecipitation followed by qPCR (n = 3). (D) CircRNA pull-down assay. The RNA-protein complexes were used to extract proteins, which were subjected to western blot analysis of E2F1 (n = 3). (E) Western blotting analyses of E2F1 levels in the whole cell lysates and the nuclear fractions of the indicated cells (n = 3). (F) Western blot analyses of nuclear E2F1 levels in circFOXO3-knockdown MLE12 cells

treated with or without 2.5% CSE for 24 h (n = 3). (G) SA-β-gal staining of cells treated with 2.5% CSE (n = 3). (H, I) Western blot analysis of E2F1, Beclin 1, p16, and p21 in cells treated with 2.5% CSE and/or 3-MA (n = 3). $^{**}P$<0.01 *vs*. NC/Con group, $^{\#\#}P$<0.01 *vs*. NC/CSE group, $^{\&\&}P$<0.01 *vs*. shcircFOXO3/CSE group.

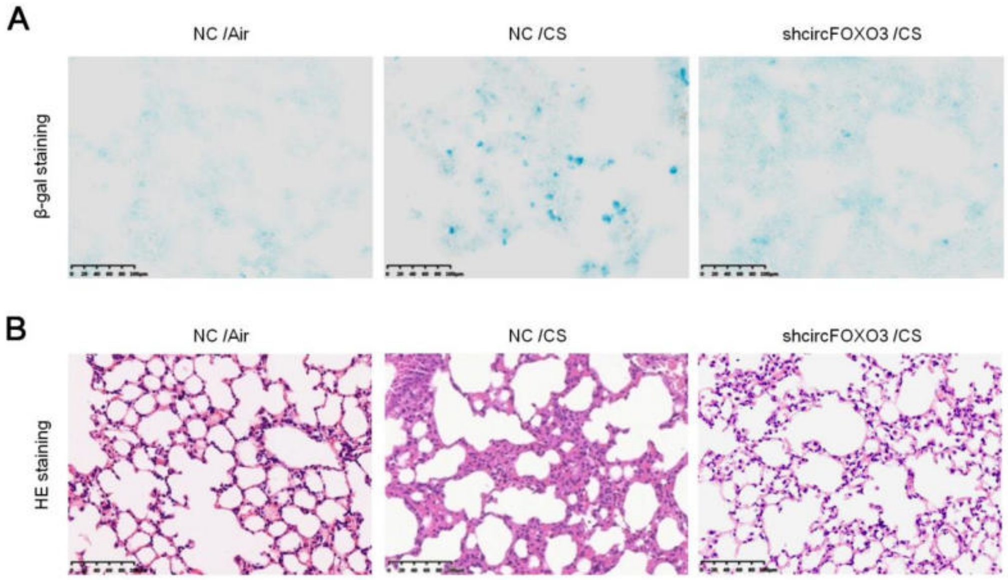

Supplementary Figure S2. CircFOXO3 knockdown protected against CS-induced AT-II cell senescence *in vivo*. β-Gal (A) and H&E (B) staining of lungs from mice exposed and not exposed to CS (n = 6).

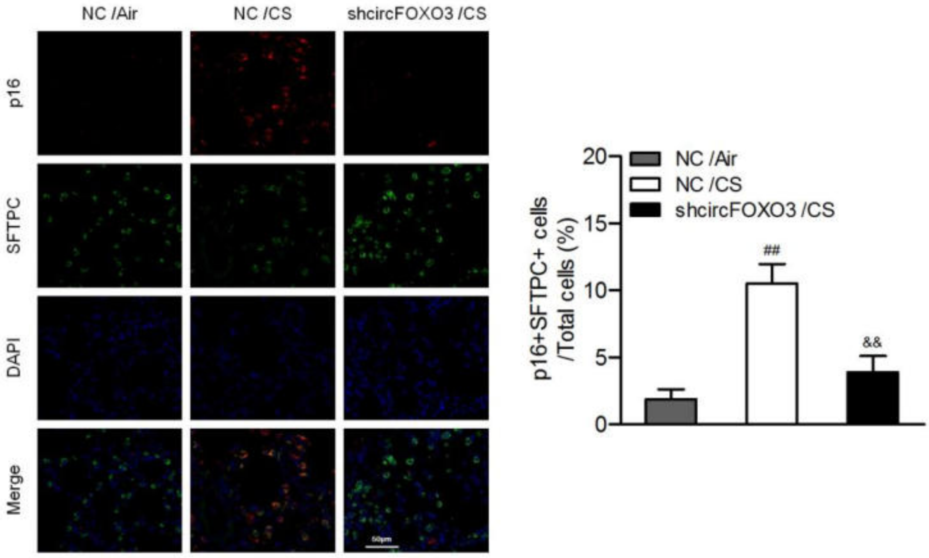

Supplementary Figure S3. Immunofluorescence microscopy to determine colocalization of p16 and SFTPC. The ratio of p16+ SFTPC+ cells to total cells is presented on the right (n = 6). ##P<0.05 *vs*. NC/Air group, &&P<0.01 *vs*. NC/CS group.

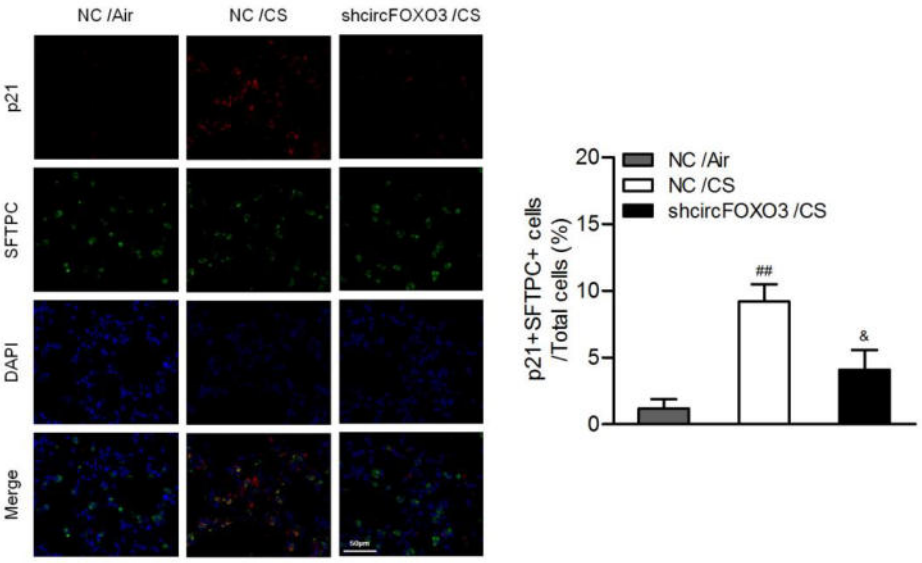

Supplementary Figure S4. Immunofluorescence microscopy to determine colocalization of p21 and SFTPC. The ratio of p21+ SFTPC+ cells to total cells is presented on the right (n = 6). $^{\#\#}P$<0.05 *vs*. NC/Air group, $^{\&}P$<0.05, $^{\&\&}P$<0.01 *vs*. NC/CS group.

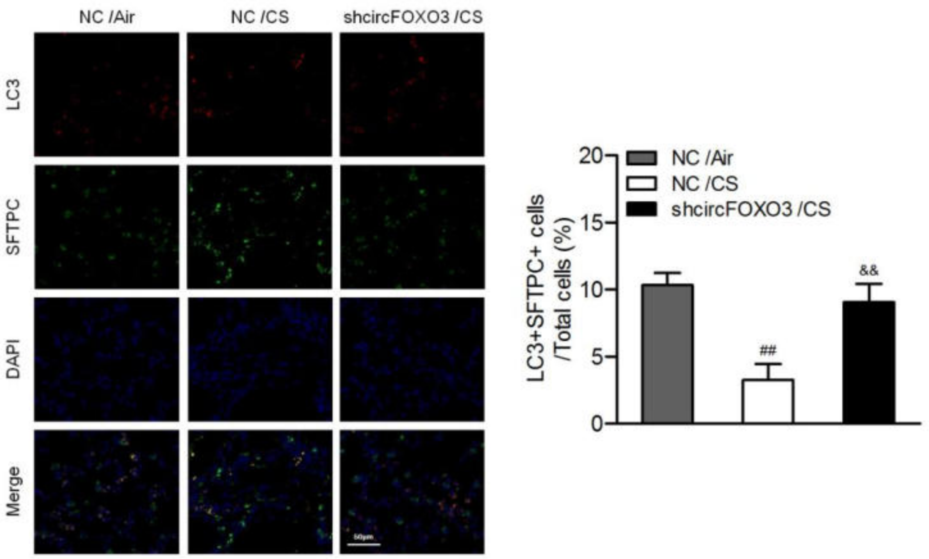

Supplementary Figure S5. Immunofluorescence microscopy to determine colocalization of LC3 and SFTPC. The ratio of LC3+ SFTPC+ cells to total cells is presented on the right (n = 6). $^{\#\#}P$<0.05 *vs*. NC/Air group, $^{\&\&}P$<0.01 *vs*. NC/CS group.

复旦大学附属中山医院动物实验伦理审查申请书

Zhongshan Hospital, Fudan University
Application for Ethical Approval for Research Involving Animals

申请日期：2022 年 2月 8 日
App. Date(YY-MM-DD):

申请人姓名<br>Name of Principal Investigator	杜春玲	申请人单位<br>Department	复旦大学附属中山医院青浦分院呼吸内科
联系人姓名<br>Contact Person	杜春玲	联系人电话<br>Contact Tel. No.	13917602796
课题名称<br>Project Title	circFOXO3 促进香烟诱导的 COPD 合并肺纤维化的机制研究		
课题性质<br>Funding Source & Number	国家自然科学基金项目		

	姓名<br>Name	职称<br>Title	实验动物从业人员上岗证号<br>Animal User Permit No.	联系电话<br>Tel.No.
参与实验人员<br>Additional Research Personnel	杜春玲	主任医师	Y20090714（湖北省实验动物学会）	13917602796
	周磊	副主任医师	复旦大学攻读研究生期间实验动物中心培训合格	18116016019
	周俊	主治医师	复旦大学攻读研究生期间实验动物中心培训合格	13301989572
	孙雨晴	主治医师	上海交通大学攻读研究生期间实验动物中心培训合格	18117509841

实验概述（包括实验目的，实验设计，对动物进行的每一步详细操作，实验结束后动物的处理包括安死术等。）

Description of Project (Please describe study objectives and experimental design; specify all procedures performed on animals, post procedure animal care, and the fate of animals including euthanasia criteria and method.)

实验目的：circFOXO3 对 CS 暴露小鼠模型肺纤维化的影响

本实验使用雄性 C57BL/6J 小鼠（6-8 周，共 40 只），FOXO3a 敲除小鼠（20 只），实验分为 6 组，每组 10 只：正常对照组（WT-Normal）、CS 暴露诱导小鼠模型组（WT-CS）、CS 暴露诱导小鼠模型组转染 circFOXO3 沉默病毒载体（WT-CS+ circFOXO3-KD）、CS 暴露诱导小鼠模型组转染空病毒载体作为对照（WT-CS+NC）、CS 暴露诱导 FOXO3 敲除小鼠模型组转染 circFOXO3 沉默病毒载体（KO-CS+ circFOXO3-KD）、CS 暴露诱导 FOXO3 敲除小鼠模型组转染空病毒载体作为对照（KO-CS+NC）。采用连续香烟烟雾暴露法建立小鼠 COPD 模型。

实验设计：

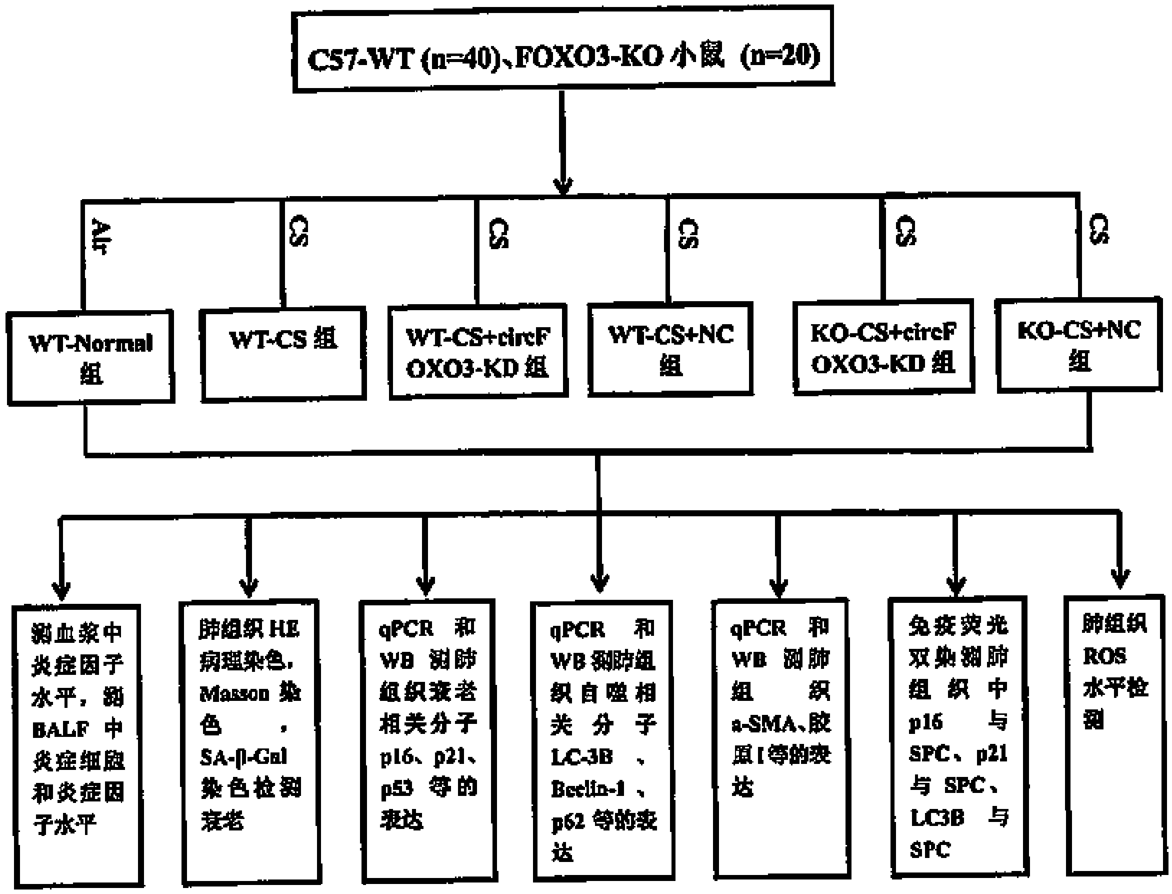

详细操作：

① CS 暴露 COPD 小鼠模型的建立：

CS组将小鼠置于自制有机玻璃箱（70×70×50cm）内，箱体侧壁各有一个通气孔，点燃5支香烟放入箱内自然燃烧，15min后打开熏烟箱顶盖，待烟雾完全散去，同时使小鼠休息30min。然后再次点燃5支香烟重复上述步骤，一天四次，一周进行五天：对照组小鼠暴露于空气中。暴露时间设定为3个月。其中circFOXO3干扰慢病毒（circFOXO3-KD）、对照（NC）和空白对照（PBS）通过尾静脉给药，每两周一次。在每一个时间点，处理相应亚组的小鼠。

小鼠肺组织和 BALF 的获得：

i.用1.5%戊巴比妥腹腔注射麻醉，然后将小鼠固定后，剖开胸腔，从右心室采血，处死小鼠。

ii. 气管上切开小口，插入改良的18G气管插管针，结扎固定。丝线结扎右侧肺门处，剪下右侧肺组织放到冻存管中，马上放到盛有液氮的容器中速冻，实验结束后-80° C保存。

iii. 小鼠左肺用500 μL PBS灌洗3次，每次回抽2遍，回抽率在80%以上认为回抽成功。支气管肺泡灌洗液（BALF），进行细胞总数与分类的计数，并进行炎症因子等相关因子的表达和分泌，然后用4%多聚甲醛固定，制成病理切片观察炎症浸润与肺气肿形成情况；取右肺用于提取mRNA和蛋白，用qRT-PCR和Western blot检测衰老、自噬及纤维化相关分子的表达。

② 小鼠肺组织病理分析

制作石蜡切片，采用苏木精染色、蒸馏水、1%盐酸酒精分化蒸馏水(20s)、蒸馏水。接着伊红染色、蒸馏水、70%无水乙醇、80%无水乙醇、90%无水乙醇、100%无水乙醇脱水，二甲苯透明进行三次；然后使用中性树胶封片，显微镜下采集图片信息。

③ Masson染色：小鼠肺组织用4%多聚甲醛固定，石蜡包埋。将连续的5 μm切片置于玻片上，并用Masson染色标准方法进行染色。通过胶原比表面积(蓝色)与总比表面积(红色)的比值来测定胶原含量。

④ SA-β-Gal染色：小鼠肺组织切冰冻切片，用碧云天染色试剂盒进行染色。

<table>
<tr><td rowspan="4">使用动物情况
Animal Requirements</td><td colspan="4">动物来源 Source　C57BL/6J 小鼠，动物中心代购
FOXO3a 敲除小鼠 上海南方模式生物科技股份有限公司</td></tr>
<tr><td>品种品系 Species or Strains
C57BL/6J</td><td colspan="2">等级 Grade
SPF 级</td><td>规格 Specifications
6-8 周</td></tr>
<tr><td colspan="4">数量 Quantity　60 只（♀　60 只；♂　只）</td></tr>
<tr><td colspan="2">计划进驻日期（Proposed Date of Commencement）
2022 年 10 月 8 日</td><td colspan="2">计划结束日期（Proposed Date of Completion）
2024 年 2 月 8 日</td></tr>
<tr><td>申请人承诺
Principal Investigator's Declaration</td><td colspan="4">本人已认真审阅此申请表所填内容，保证所填内容真实可靠，并将严格遵守上述实验方案。(I confirm that I will ensure that the requirements for the treatment of the animals as detailed in this application and as approved by the Animal Ethics Committee will be met during the course of the project.）

签名（Signature）　[illegible]　　日期（Date）　2022年 2月 11日</td></tr>
</table>

审查项目<br>Ethical<br>Considerations	1. 该项目的科学重要性和必要性，期望的科学获益。（The significances, necessity and the expected scientific benefits of the proposal work.）<br>2. 所使用的动物品种、等级、规格是否合适，使用数量的计算依据。（The species, strains, grade, specification and number of the animals to be used should be justified.）<br>3. 能否通过改良设计方案替代或减少使用所用动物。(Rational for animal use should be justified, including the alternatives to animal use, a refined study design to replace or reduce animal number to be used..)<br>4. 实验操作中是否善待动物，包括合理的实验终点，麻醉方案，不麻醉的理由及减少相应动物痛苦的措施，实验结束动物的处理，动物安乐死方案等。（Appropriate animal care and handling throughout the experiment, including a scientific sound endpoint; anesthetics, analgesics, sedatives or tranquilizers that are to be used; explanation for any procedure cause unrelieved pain or distress; disposition of animals at end of study; and euthanasia criteria and method.）<br>5. 是否使用对人体或环境有害试剂，有潜在感染性试剂，放射性物质；是否使用遗传修饰试剂；是否进行遗传操作。相应的防护措施。（Are the materials to be used harmful or toxic? Are there any radioactive agents, infectious agents, genetic modified agents, and the genetic manipulation to be used in the experiment? If yes, the safety measures should be specified.）<br>6. 是否使用转基因动物，来源是否符合国家规定，是否进行免疫隔离。(Are genetic modified animals to be used in the experiments? IF yes, the source should be valid and the animals should be quarantined.)
实验动物保护、福利、伦理小组意见<br>Comments, Provisos or Reservations of Animal Ethics Committee<br><br>复旦大学附属中山医院 动物伦理委员会<br><br>代表签名：<br>Name of Ethics Committee Representative<br><br>Date(YY-MM-DD): 2022 年 2 月 22 日	

© 2026 Zhou X. et al.
